# Changes in the inflammatory and oxidative stress markers during a single hemodialysis session in patients with chronic kidney disease

**DOI:** 10.1080/0886022X.2018.1487857

**Published:** 2018-10-02

**Authors:** B. Sangeetha Lakshmi, N. Harini Devi, M. M. Suchitra, P. V. L. N. Srinivasa Rao, V. Siva Kumar

**Affiliations:** a Department of Nephrology, Sri Venkateswara Institute of Medical Sciences, Tirupati, India;; b Department of Biochemistry, Sri Venkateswara Institute of Medical Sciences, Tirupati, India

**Keywords:** Cardiovascular disease, hemodialysis, inflammation, oxidative stress; Pentraxin-3

## Abstract

**Background**: Cardiovascular disease (CVD) is a common cause of morbidity and mortality in end-stage renal disease (ESRD) patients on hemodialysis (HD) among whom it is 5–20 times higher than in the general population. Some of the nontraditional risk factors such as oxidative stress and inflammation are related to the progress of CVD in HD patients. Several, but not all studies, reported that inflammatory and oxidative stress markers are increased during a single session of HD, mimicking changes that occur during acute immune activation. This study was taken up to evaluate the changes in the inflammatory and oxidative stress markers during a single HD session in patients with chronic kidney disease.

**Methods:** Twenty-five ESRD patients on maintenance HD and 25 controls were included in the study. Blood samples were obtained from the patients before starting of hemodialysis (pre-HD) and after completion of hemodialysis (post-HD). The changes in serum Pentraxin-3, hs-CRP, malondialdehyde (MDA) and ferric reducing ability of plasma (FRAP) levels were measured in pre- and post-HD ESRD patients and compared with healthy control group.

**Results:** This study found increased levels of Pentraxin-3, hs-CRP, MDA, and decreased level of FRAP in HD patients compared to controls.

**Conclusions:** Hemodialysis procedure contributes to inflammation and oxidative stress.

## Introduction

Cardiovascular disease (CVD) is extremely prevalent in maintenance hemodialysis (HD) patients and accounts for approximately half of deaths in chronic kidney disease (CKD) [1].

The annual mortality in end-stage renal disease (ESRD) patients is about 10- to 20-fold higher than that in the general population [2]. These patients show a high cardiovascular morbidity and mortality that might be induced by inflammation and oxidative stress. There are several risk factors for developing CVD in CKD patients, which are categorized into traditional and nontraditional risk factors. Traditional risk factors commonly present in the CKD population such as older age, hyperlipidemia, hypertension and diabetes mellitus cannot explain the unacceptable high prevalence and incidence of CVD in these patients. Hence, nontraditional risk factors such as inflammation, oxidative stress, malnutrition, anemia and vascular calcification have been an area of intense investigation, in this population and are being recognized [3]. Inflammation in ESRD patients on HD is associated with malnutrition and CVD that results in poor clinical outcome [4]. Various events are common causes of inflammation, such as periodontal disease, bioincompatible dialysis membranes, unpure dialysate, and vascular access [5]. Bioincompatible dialysis membranes were more common with earlier membranes, especially cuprophan membranes, which induced inflammation. Synthetic membranes, used today, are far more biocompatible [6]. In the late 1990s, C-reactive protein (CRP) was acknowledged as powerful predictor of cardiovascular death and overall mortality in HD and peritoneal dialysis (PD) patients. Interleukin-6 (IL-6) was also shown to be a strong predictor of mortality in HD patients. IL-6 and CRP are measurable markers of inflammation. It was reported that given the strong correlation between high-sensitive C-reactive protein (hs-CRP) and IL-6, the hs-CRP analysis may suffice in the clinical situation [7]. Boehme et al. first described that the Pentraxin (PTX3) levels in HD patients with ESRD are higher than healthy subjects or ESRD patients without receiving HD [8]. Pentraxin as well as CRP, belongs to the Pentraxin family of proteins, is an acute phase reactant involved in pathogen recognition, complement activation and regulation. The first cloned long Pentraxin is Pentraxin 3, PTX3 has a bigger molecular size (40.6 kDa) compared to CRP (21.5 kDa) and has a unique PTX3 domain not found in CRP or SAP [9]. Moreover, PTX3, in contrast to the liver-produced CRP, is produced by different cell types, including endothelial cells, phagocytes, dendritic cells, smooth muscle cells, fibroblasts, and adipocytes in response to primary local inflammation and innate immunity and may directly reflect the inflammatory status [10]. Plasma PTX3 is considered as an inflammatory marker of endothelial dysfunction and is also linked to increasing cardiovascular mortality risk [11]. PTX3 is elevated in dialysis patients compared to healthy controls and reflects endothelial dysfunction associated with CVD and mortality risk [12].

Increased oxidative stress occurs in HD patients and is dependent on many factors such as aging, loss of residual renal function, uremic conditions, and those receiving regular HD [13]. Peroxidation of membrane polyunsaturated fatty acids by free radicals produces some molecules such as malondialdehyde (MDA) that could be useful as an indicator for assessing oxidative damage. Significant elevation of serum MDA levels is observed in CKD on HD patients with CVD, compared with those without CVD, shows the relationship between oxidative stress and the development of atherosclerosis in these patients [14]. Hence, the present study aimed to estimate changes in the inflammatory and oxidative stress markers during a single HD session in patients with CKD.

## Materials and methods

Twenty five (25) patients with ESRD on maintenance HD in the Department of Nephrology, Sri Venkateswara Institute of Medical Sciences and 25 age and sex-matched healthy subjects as controls were recruited in the present study during the period May 2015 to October 2015. Institutional Ethics Committee approval and written informed consent were obtained from all the participants before the conduct of study-related procedure. ESRD patients on HD received regular HD treatment three times a week (4–5 h per session) through arteriovenous fistulas. Twenty five healthy individuals among the patients’ relatives and hospital staff, who were non-smokers, non-diabetics as per ADA criteria, non-hypertensive as per Joint National Committee (JNC) VIII were included as control group. Subjects on HD treatment less than three months duration, age younger than 16 years, presence of HIV or Hepatitis B/C infection, chronic inflammatory (malignancy, liver disease) and infective conditions, pregnant women and unwilling patients were excluded. In our maintenance HD program, we perform low-flux conventional intermittent thrice weekly HD as maintenance renal replacement therapy using Fresenius HD machine, polysulfone membrane, and bicarbonate dialysate, wherein the blood flow rate is between 200 and 300 mL/min and dialysate flow around 500 mL/min for a total duration of 4 h. Water for the dialysate was purified by reverse osmosis. Anticoagulation was performed with 2000 IU of heparin at the start of dialysis followed by continuous administration at a rate of 500–1000 IU/h. The pre-HD sample was collected from afferent fistula needle inserted to start the dialysis before connecting it to the dialyzer. Post-HD sample was collected after 4 h from the same port just before the termination of the dialysis procedure.

### Laboratory analysis

Six milliliters of venous blood sample just before commencing dialysis (pre-HD) and at the termination of dialysis (post-HD) was collected in additive free tubes. The blood samples were allowed to stand for 30 min, centrifuged at 3000 rpm for 15 min and the separated serum was stored at −80 °C until further analysis. Serum urea, creatinine, uric acid, total cholesterol, triglycerides, HDL, calcium, phosphorus, total protein, and albumin were estimated using commercial kits. Low density lipoprotein (LDL) and very low density lipoprotein (VLDL) were calculated by Freidewald’s equation [15]. hs-CRP was estimated by immunoturbidimetry method by commercial kits. All the above parameters were analyzed on clinical chemistry Auto analyzer Beckman Coulter DXC 600 Synchron (‎Brea, CA). Plasma MDA and ferric reducing ability of plasma (FRAP) were estimated by spectrophotometric method using Perkin-Elmer spectrophotometer, Lambda 25 UV/VIS Spectrophotometer (Waltham, MA) [16,17]. Pentraxin-3 was estimated by ELISA method using the commercially available human Pentraxin ELISA kit (Hycult, Beutelsbach, Germany).

### Correction of special parameters

Post dialysis samples of serum Pentraxin 3 and serum hs-CRP were corrected to ultrafiltration (UF) by using the following UF formula: 1 + ΔBW/0.2 × BW [18], where BW is the change in the bodyweight during HD and that the extracellular volume is 20% of the post-dialysis BW, assuming linear UF during HD.

Serum MDA was corrected to serum creatinine and was expressed as μmol/mg of creatinine.

### Statistical analysis

As the sample size was small (*n* = 25), data were represented as median and IQR. Categorical values were presented as numbers and percent. Comparisons between two groups for continuous variables were assessed with Mann–Whitney’s *U*-test, Wilcoxon’s sum test and for categorical values with chi-square (*χ*^2^) test, as appropriate. Differences among three groups were analyzed by the Kruskal–Wallis analysis. Spearman’s rank correlation was used to determine correlations of PTX3 concentration with other variables. All statistical analyses were performed with SPSS (version 16.0, SPSS Inc., Chicago, IL). A *p* value of <.05 was considered significant.

## Results

All patients of the study are from the Department of Nephrology, Sri Venkateswara Institute of Medical Sciences. Twenty five ESRD patients on maintenance HD and 25 controls were included in the study. The general characteristics of 25 HD patients and 25 controls are shown in Table 1. The median and IQR of the baseline routine biochemical parameters of the controls and patients of both pre-HD and post-HD groups are shown in Table 2. There was significant increase in serum urea, creatinine, cholesterol, potassium, phosphorus, and uric acid levels in group 1 (pre-HD) when compared to the group 3 (controls) (*p* < .001). There was significant decrease in serum LDL, sodium, albumin, and calcium in pre-HD group when compared to the controls (*p* < .05). Serum total proteins, triglycerides, HDL, and VLDL did not show any significant difference in patients when compared to the controls. Statistical analysis using Mann–Whitney’s *U*-test of the routine biochemical parameters of the group 1 (pre-HD) and group 3 (controls) are shown in Table 3. Mean rank was calculated and observed that there was significant increase in serum urea, creatinine, cholesterol, potassium, phosphorus, and uric acid levels in group 1 (pre-HD) when compared to the group 3 (controls) (*p* < .001). There was significant decrease in serum LDL and sodium in pre-HD group when compared to the controls (*p* < .05). Serum total proteins, albumin, triglycerides, HDL, VLDL, and calcium did not show any significant difference in patients when compared to the controls. Statistical analysis using Wilcoxon’s sum test of the routine biochemical parameters of the group 1 (pre-HD) and group 2 (post-HD) are shown in Table 4. Based on the *Z* score, it was showed that there was significant difference in serum urea, creatinine, cholesterol, LDL, sodium, potassium, calcium, phosphorus, and uric acid levels. Serum total proteins, albumin, triglycerides, HDL, and VLDL levels did not show any significant difference in group 1 (pre-HD) when compared to group 2 (post-HD). The median and IQR of the inflammatory (Pentraxin-3 and hs-CRP) and oxidative stress markers (MDA and FRAP) of the controls and the patients of both pre-HD and post-HD are shown in Table 5. Serum Pentraxin-3, hs-CRP, MDA, and FRAP were significantly increased in ESRD patients on HD when compared to controls (*p* = .001). Serum Pentraxin-3 and hs-CRP levels were corrected to UF. The median and IQR of the special biochemical parameters of the group 1 (pre-HD) and group 2 (post-HD) after correction are shown in Table 6. There was significant elevation of serum Pentraxin-3 and hs-CRP after a single session of HD (*p* < .001). Malondialdehyde decreased after a single session of HD, but correcting MDA for creatinine, we found a significant increase in MDA after a single HD session (*p* < .001). There was a significant decrease in post-FRAP levels (*p* = .004). In the present study, Pentraxin 3 was correlated with the other variables and we observed statistically significant positive correlations among PTX3 with hs-CRP (*r* = 0.283, *p* = .047) and statistically significant negative correlations with FRAP (*r*= −0.429, *p* = .002) but no association with MDA (*r*= −0.127, *p* = .380 NS).

**Table 1.** General characteristics of the subjects in the present study.

Parameter	Controls	Hemodialysis (HD) patients	*p* Value
Number (*n*)	25	25	–
Age (mean ± SD) (years)	50.00 (26.00–70.00)	50.00 (26.50–70.30)	.721*
Males:Females	14:11	14:11	–
Body mass index (kg/m^2^)	24.00 (18.00–29.00)	23.00 (19.00–27.00)	.449*

*n*: sample size; SD: standard deviation.

*NS: not significant at the .05 probability level.

**Table 2.** Baseline routine biochemical parameters in controls and patients (pre-HD and post-HD values).

Parameter	Patients			
	Pre-HD (*n* = 25)	Post-HD (*n* = 25)	Controls (*n* = 25)	*p* Value
	(group 1)	(group 2)	(group 3)	
Urea (mg/dL)	96.00 (46.00–147.00)	39.00 (21.00–78.00)	22.00 (14.00–50.00)	<.001*
Creatinine (mg/dL)	7.56 (4.22–13.25)	3.78 (1.59–11.50)	0.82 (0.58–1.60)	<.001*
Albumin (g/dL)	3.90 (3.00–4.40)	4.00 (2.90–4.70)	4.1 (3.0–4.7)	.030†
Total proteins (g/dL)	7.40 (5.90–9.30)	7.70 (5.70–9.70)	7.5 (7.0–8.4)	.326†
Triglycerides (mg/dL)	124.00 (60.00–285.00)	118.00 (51.00–287.00)	131.00 (90.00–268.00)	.363†
Cholesterol (mg/dL)	175.00 (155.00–222.00)	154.00 (96.00–234.00)	144 (88.00–216)	.003*
HDL (mg/dL)	40.00 (26.00–50.00)	41.00 (29.00–60.00)	40.00 (31.00–53.00)	.688†
LDL (mg/dL)	70.00 (33.00–139.00)	87.00 (42.00–158.00)	108.00 (86.00–150.00)	<.001*
VLDL (mg/dL)	25.00 (10.00–57.00)	24.00 (10.00–57.00)	26.00 (18.00–54.00)	.614†
Sodium (mmol/L)	128.00 (122.00–133.00)	129.00 (123.00–135.00)	137 (130–143)	<.001*
Potassium (mmol/L)	4.40 (3.20–5.60)	2.90 (2.20–4.80)	3.8 (3.1–4.5)	<.001*
Calcium (mg/dL)	8.80 (7.30–10.30)	9.70 (7.90–10.80)	9.1 (8.5–9.9)	<.001*
Phosphorus (mg/dL)	4.50 (2.80–12.20)	3.00 (1.30–7.40)	3.9 (2.9–4.2)	.001*
Uric acid (mg/dL)	5.10 (2.90–8. 80)	2.10 (1.20–5.40)	4.00 (2.30–5.60)	<.001*

HDL: high density lipoprotein; LDL: low density lipoprotein; VLDL: very low density lipoprotein.

*Significant at the .05 probability level.

†NS: not significant at the .05 probability level.

**Table 3.** Routine biochemical parameters: group 1 versus group 3: unpaired *t* test.

Parameter	Mean rank	Mean rank	*p* Value
	Pre-HD (*n* = 25) (group 1)	Controls (*n* = 25) (group 3)	
Urea (mg/dL)	37.92	13.08	<.001*
Creatinine (mg/dL)	38.00	13.00	<.001*
Albumin (g/dL)	21.96	29.04	.085†
Total proteins (g/dL)	22.90	28.10	.206†
Triglycerides (mg/dL)	23.64	27.36	.367†
Cholesterol (mg/dL)	33.40	17.60	<.001*
HDL (mg/dL)	25.02	25.98	.816†
LDL (mg/dL)	15.90	35.10	<.001*
VLDL (mg/dL)	23.42	27.58	.312†
Sodium (mmol/L)	13.58	37.42	<.001*
Potassium (mmol/L)	33.40	17.60	<.001*
Calcium (mg/dL)	22.12	28.88	.100†
Phosphorus (mg/dL)	32.80	18.20	<.001*
Uric acid (mg/dL)	33.34	17.66	<.001*

HDL: high density lipoprotein; LDL: low density lipoprotein; VLDL: very low density lipoprotein.

*Significant at the .05 probability level.

†NS: not significant at the .05 probability level.

**Table 4.** Routine biochemical parameters: group 1 versus group 2: paired *t* test.

Parameter	Pre-HD (*n* = 25)	Post-HD (*n* = 25)	*Z* Score	*p* Value
	(group 1)	(group 2)		
Urea (mg/dL)	96.00 (46.00–147.00)	39.00 (21.00–78.00)	–4.320	<.001*
Creatinine (mg/dL)	7.56 (4.22–13.25)	3.78 (1.59–11.50)	–4.372	<.001*
Albumin (g/dL)	3.90 (3.00–4.40)	4.00 (2.90–4.70)	1.467	.142†
Total proteins (g/dL)	7.40 (5.90–9.30)	7.70 (5.70–9.70)	1.702	.089†
Triglycerides (mg/dL)	124.00 (60.00–285.00)	118.00 (51.00–287.00)	1.063	.288†
Cholesterol (mg/dL)	175.00 (155.00–222.00)	154.00 (96.00–234.00)	–2.597	.009*
HDL (mg/dL)	40.00 (26.00–50.00)	41.00 (29.00–60.00)	1.333	.183†
LDL (mg/dL)	70.00 (33.00–139.00)	87.00 (42.00–158.00)	2.401	.016*
VLDL (mg/dL)	25.00 (10.00–57.00)	24.00 (10.00–57.00)	–0.579	.562†
Sodium (mmol/L)	128.00 (122.00–133.00)	129.00 (123.00–135.00)	–2.427	.015*
Potassium (mmol/L)	4.40 (3.20–5.60)	2.90 (2.20–4.80)	–4.376	<.001*
Calcium (mg/dL)	8.80 (7.30–10.30)	9.70 (7.90–10.80)	3.502	<.001*
Phosphorus (mg/dL)	4.50 (2.80–12.20)	3.00 (1.30–7.40)	–4.153	<.001*
Uric acid (mg/dL)	5.10 (2.90–8. 80)	2.10 (1.20–5.40)	4.288	<.001*

HDL: high density lipoprotein; LDL: low density lipoprotein; VLDL: very low density lipoprotein.

*Significant at the .05 probability level.

†NS: not significant at the .05 probability level.

**Table 5.** Baseline special biochemical parameters in controls and patients (pre-HD and post-HD values).

Parameter	Pre-HD (*n* = 25)	Post-HD (*n* = 25)	Controls (*n* = 25)	*p* Value
	(group 1)	(group 2)	(group 3)	
Pentraxin (ng/mL)	1.96 (1.62–2.72)	1.75 (1.40–2.30)(uncorrected to ultrafiltration)	0.38 (0.24–0.65)	<.001*
hs-CRP (mg/dL)	1.80 (1.10–5.80)	1.85 (1.12–5.90)(uncorrected to ultrafiltration)	0.33 (0.21–1.00)	<.001*
MDA (μmol/L)	3.98 (2.33–6.45)	3.37 (2.10–6.20)	1.20 (0.55–1.87)	<.001*
FRAP (mmol/L)	0.80 (0.51–1.18)	0.40 (0.25–0.82)	0.57 (0.41–0.95)	.001*

MDA: malondialdehyde; hs-CRP: high-sensitive C-reactive protein; FRAP: ferric reducing ability of plasma.

*Significant at the .05 probability level.

**Table 6.** Special biochemical parameters: group 1 versus group 2: paired *t* test.

Parameter	Pre-HD (*n* = 25)	Post-HD (*n* = 25)	*p* Value
	(group 1)	(group 2)	
Pentraxin (ng/mL)	1.96 (1.62–2.72)	2.05 (1.71–2.63)(corrected to ultrafiltration)	<.001*
hs-CRP (mg/dL)	1.80 (1.10–5.80)	2.18 (1.40–6.17)(corrected to ultrafiltration)	<.001*
MDA/corrected for serum creatinine (μmol/mg)	3.75 (2.15–6.18)	3.76 (2.31–6.57)	<.001*
FRAP (mmol/L)	0.80 (0.51–1.18)	0.73 (0.36–1.19)	.004

MDA: malondialdehyde; hs-CRP: high-sensitive C-reactive protein; FRAP: ferric reducing ability of plasma.

*Significant at the .05 probability level.

## Discussion

**Figure 1.**
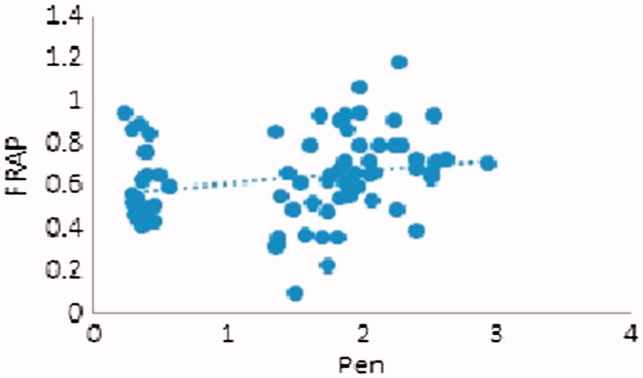
Scatter plot showing association of Pentraxin-3 and FRAP.

Inflammation is a common feature of ESRD patients with or without maintenance HD [19]. Increase in inflammatory markers in dialysis patients has been shown to be associated with a 35% higher risk of mortality [20]. The changes in the inflammatory markers (serum Pentraxin-3 and hs-CRP) and the oxidative stress markers (serum MDA and FRAP) during a single HD session were studied. The subjects in our study had higher level of PTX3 in ESRD patients on HD when compared to healthy controls. A study by Tong et al. reported that patients with higher PTX3 levels had a higher cardiovascular mortality with hazard ratio 1.83 when comparing patients within the highest PTX3 tertile with patients within the other two tertiles [21]. The present study also showed a significant elevation of serum Pentraxin-3 after a single session of HD, suggesting the role of dialysis perse in the process of inflammation. This was also well comparable to previous studies as reported by Malaponte et al. [22]. The hemoconcentration due to UF should be taken into account in studies measuring peptide concentrations during HD. In the present study, serum Pentraxin-3 and hs-CRP levels were corrected for net UF. Our study also showed that HD patients have higher plasma PTX3 concentrations than normal individuals along with increased hs-CRP levels. Traditional assays for CRP are not sensitive for measuring the lower serum values associated with inflammatory process. The newer hs-CRP assays are capable of measuring serum CRP to below 0.1 mg/L. Korevaar et al. in their study observed an increase in CRP in 26% of their patients after adjusting for volume changes during HD [20]. The result that significantly increased PTX3 levels after a single HD session in the present study suggested HD procedure itself could induce inflammation. Various dialysis-related factors such as dialyzer membrane bioincompatibility, type of vascular access and dialysate contamination may promote a persistent, low-grade inflammatory response in these patients. Due to its extrahepatic synthesis, the PTX3 level is believed to be a true independent indicator of endothelial cell injury in contrast to CRP [19]. Furthermore, in response to a stimulus, PTX3 increases in plasma more rapidly than CRP [23]. Hence, it may be considered as a novel marker to evaluate the inflammatory response in ESRD patients on dialysis. PTX-3 is described as inflammatory molecule that belongs to the same family as CRP [24]. In agreement with, and confirming, previous findings, the current study reports strong association between PTX-3 and FRAP in HD patients as shown in Figure 1. Oxidative stress poses a serious threat to the cardiovascular outcome in end stage renal disease. In our study, MDA was significantly increased in end stage renal disease patients on dialysis when compared to controls. These findings are supported by Hacişevki who reported multiple factors that influence oxidative stress in HD therapy such as increase in the production of agents from oxidative metabolism and a decrease in antioxidant defenses, the use of low biocompatible membranes and purity of dialysis water [25]. Malondialdehyde decreased after a single session of HD, but it was not significant. Malondialdehyde being a small water soluble molecule can diffuse across dialysis membranes. Taking into consideration of the clearance of MDA during dialysis, the ratio of MDA and creatinine was likely to give a better picture about MDA during dialysis. After correcting MDA for creatinine, we found a significant increase in MDA levels from pre-dialysis to the post-dialysis indicating that the increase in MDA during HD was due to its increased production consequent to oxidative stress, rather than getting cleared by dialysis [26]. This increase in post-HD MDA was an indicator of the presence of oxidative stress during the dialysis session [27]. In the present study, there was significant increase in FRAP in end stage renal disease patients on dialysis when compared to controls. This was in accordance with many studies who observed altered nonenzymatic and enzymatic antioxidants in ESRD patients on PD and on HD [28]. We found a significant decrease in post-HD FRAP compared with predialysis levels. Uric acid, which has strong reducing and antioxidant properties but increased levels of uric acid, was observed as a potential risk factor for CVD [29]. Uric acid at higher concentrations was found to behave as a pro-oxidant under conditions of oxidative stress, especially in the presence of deficiencies in antioxidant systems. Uric acid was known to be cleared by HD as evidenced by a decrease in uric acid levels when compared to pre-HD in the present study. In the FRAP assay, uric acid contributes to a major portion of the FRAP values, which is about 60%. It had been reported that FRAP correlated significantly with serum uric acid and bilirubin levels in HD patients and controls [30]. In our study, there was significant decrease in post-HD uric acid levels when compared to predialysis levels, hence a change in post FRAP levels was observed.

To conclude, the present study findings suggest that during a single session of HD, there is increase in inflammation as observed by statistically significant increase in serum Pentraxin-3 and hs-CRP and increase in oxidative stress as observed by a statistically significant increase in serum MDA. These findings suggest that changes in inflammatory and oxidative stress biomarkers occur during a single HD session but further studies are required for monitoring the intradialytic inflammatory response during HD and also follow up of association of CVD risk-related biomarkers with the cardiovascular outcomes in ESRD patients is more beneficial.

## References

[1] Parfrey PS, Foley RN The clinical epidemiology of cardiac disease in chronic renal failure. J Am Soc Nephrol. 1999;10:1606–1615. 10405218 10.1681/ASN.V1071606

[2] Foley RN, Parfrey PS, Sarnak MJ Clinical epidemiology of cardiovascular disease in chronic renal disease. Am J Kidney Dis. 1998;32:S112–S119. 9820470 10.1053/ajkd.1998.v32.pm9820470

[3] Stenvinkel P, Carrero JJ, Axelsson J, et al. Emerging biomarkers for evaluating cardiovascular risk in the chronic kidney disease patient: how do new pieces fit into the uremic puzzle? Clin J Am Soc Nephrol. 2008;3:505–521. 18184879 10.2215/CJN.03670807 PMC6631093

[4] Stenvinkel P Inflammation in end-stage renal failure: could it be treated? Nephrol Dialysis Transplant. 2002;17:33–40. 10.1093/ndt/17.suppl_8.33 12147775

[5] Raj DS, Carrero JJ, Shah VO, et al. Soluble CD14 levels, interleukin 6, and mortality among prevalent hemodialysis patients. Am J Kidney Dis. 2009;54:1072–1080. 19733948 10.1053/j.ajkd.2009.06.022 PMC2787958

[6] Qureshi AR, Alvestrand A, Divino-Filho JC, et al. Inflammation, malnutrition, and cardiac disease as predictors of mortality in hemodialysis patients. J Am Soc Nephrol. 2002;13:28–36. 11792759

[7] Meuwese CL, Halbesma N, Stenvinkel P, et al. Variations in C-reactive protein during a single haemodialysis session do not associate with mortality. Nephrol Dial Transplant. 2010;25:3717–3723. 20484301 10.1093/ndt/gfq273

[8] Boehme M, Kaehne F, Kuehne A, Bernhard W, Schroder M, Pommer W, et al. Pentraxin 3 is elevated in haemodialysis patients and is associated with cardiovascular disease. Nephrol Dial Transplant. 2007;22:2224–29. 17496115 10.1093/ndt/gfl747

[9] Norata GD, Garlanda C, Catapano AL The long pentraxin PTX3: a modulator of the immunoinflammatory response in atherosclerosis and cardiovascular diseases. Trends Cardiovasc Med. 2010;20:35–40. 20656213 10.1016/j.tcm.2010.03.005

[10] Stenvinkel P, Heimburger O, Lindholm B, et al. Are there two types of malnutrition in chronic renal failure? Evidence for relationships between malnutrition, inflammation and atherosclerosis (MIA syndrome). Nephrol Dialysis Transplant. 2000;15:953–960. 10.1093/ndt/15.7.953 10862630

[11] Fazzini F, Peri G, Doni A, et al. PTX3 in small-vessel vasculitides: an independent indicator of disease activity produced at sites of inflammation. Arthritis Rheum. 2001;44:2841–2850. 11762945 10.1002/1529-0131(200112)44:12<2841::aid-art472>3.0.co;2-6

[12] Boehme M, Kaehne F, Kuehne A, et al. Pentraxin 3 is elevated in haemodialysis patients and is associated with cardiovascular disease. Nephrol Dial Transplant. 2007;22:2224–2229. 17496115 10.1093/ndt/gfl747

[13] Witko-Sarsat V, Nguyen-Khoa T, Jungers P, et al. Advanced oxidation protein products as a novel molecular basis of oxidative stress in uraemia. Nephrol Dial Transplant. 1999;14:76–78. 10.1093/ndt/14.suppl_1.76 10048460

[14] Boaz M, Matas Z, Biro A, et al. Serum malondialdehyde and prevalent cardiovascular disease in hemodialysis. Kidney Int. 1999;56:1078–1083. 10469377 10.1046/j.1523-1755.1999.00613.x

[15] Friedewald WT, Levy RI, Fredrickson DS Estimation of the concentration of low-density lipoprotein cholesterol in plasma, without use of the preparative ultracentrifuge. Clin Chem. 1972;18:499–502. 4337382

[16] Ohkawa H, Ohishi N, Yagi K Assay for lipid peroxides in animal tissues by thiobarbituric acid reaction. Anal Biochem. 1979;95:351–358. 36810 10.1016/0003-2697(79)90738-3

[17] Benzie IF, Strain JJ The ferric reducing ability of plasma (FRAP) as a measure of “antioxidant power”: the FRAP assay. Anal Biochem. 1996;239:70–76. 8660627 10.1006/abio.1996.0292

[18] Bergstrom J, Wehle B No change in corrected beta 2-microglobulin concentration after cuprophane haemodialysis. Lancet. 1987;329:628–629. 10.1016/s0140-6736(87)90266-2 2881162

[19] Peri G, Introna M, Corradi D, et al. PTX3, a prototypical long pentraxin, is an early indicator of acute myocardial infarction in humans. Circulation. 2000;102:636–641. 10931803 10.1161/01.cir.102.6.636

[20] Korevaar JC, Van manen JG, Dekker FW, Waart DR, Elisabeth W. Boeschoten EW, et al. Effect of an increase in c-reactive protein level during a hemodialysis session on mortality. J Am Soc Nephrol. 2004;15:2916–22. 15504945 10.1097/01.ASN.0000143744.72664.66

[21] Tong M, Carrero JJ, Qureshi AR, et al. Plasma pentraxin 3 in patients with chronic kidney disease: associations with renal function, protein-energy wasting, cardiovascular disease, and mortality. Clin J Am Soc Nephrol. 2007;2:889–897. 17702732 10.2215/CJN.00870207

[22] Malaponte G, Libra M, Bevelacqua Y, et al. Inflammatory status in patients with chronic renal failure: the role of PTX3 and pro-inflammatory cytokines. Int J Mol Med. 2007;20:471–481. 17786277

[23] Matsubara K, Stenvinkel P, Qureshi AR, et al. Inflammation modifies the association of osteoprotegerin with mortality in chronic kidney disease. J Nephrol. 2009;22:774–782. 19967657

[24] Bottazzi B, Vouret-Craviari V, Bastone A, et al. Multimer formation and ligand recognition by the long pentraxin PTX3. Similarities and differences with the short pentraxins C-reactive protein and serum amyloid P component. J Biol Chem. 1997;272:32817–32823. 9407058 10.1074/jbc.272.52.32817

[25] Hacişevki A Effect of haemodialysis on oxidative stress in patients with chronic renal failure. J Fac Pharm Ankara. 2008;37:91–100.

[26] Ramakrishna P, Reddy EP, Suchitra MM, et al. Effect of reuse of polysulfone membrane on oxidative stress during haemodialysis. Indian J Nephrol. 2012;22:200–205. 23087556 10.4103/0971-4065.98758 PMC3459525

[27] Reddy PE, Suchitra MM, Reddy SV, et al. Ferric reducing ability of plasma and lipid peroxidation in haemodialysis patients: intradialytic changes. Int J Nephrol Urol. 2010;2:414–421.

[28] Ansarihadipour H, Dorostkar H Comparison of plasma oxidative biomarkers and conformational modifications of hemoglobin in patients with diabetes on hemodialysis. Iran Red Crescent Med J. 2014;16:e22045. 25763223 10.5812/ircmj.22045 PMC4329937

[29] Reddy PE, Suchitra MM, Bitla AR, et al. Antioxidant enzymes status in South Indian haemodialysis patients. Int J Biol Med Res. 2011;2:682–687.

[30] Gerardi G, Usberti M, Martini G, et al. Plasma total anti-oxidant capacity in hemodialyzed patients and its relationships to other biomarkers of oxidative stress and lipid peroxidation. Clin Chem Lab Med. 2002;40:104–110. 11939481 10.1515/CCLM.2002.019

